# Supplementary material for: 3D models of fungal chromosomes to enhance visual integration of omics data
Source: NAR Genom Bioinform. 2023 Dec 5;5(4):lqad104. doi: 10.1093/nargab/lqad104 (PMC10696920; doi:10.1093/nargab/lqad104)
Supplement: lqad104_supplemental_files [file lqad104_supplemental_files.zip › Supplementary_Figures_revised.pdf]

3D models of fungal chromosomes to enhance visual integration of omics data

Thibault Poinsignon et al.

Supplementary figures

Neurospora crassa	Galazka *et al.* (2016) – Figure 6 **(*)** ; Rodriguez *et al.* (2022) – Figure 7
Saccharomyces cerevisiae	Duan *et al.* (2010) – Figure 5 **(*)** ; Tokuda *et al.* (2012) – Figure 2 **(*)**
Schizosaccharomyces pombe	Tanizawa *et al.* (2010) – Figure 4 **(*)** ; Grand *et al.* (2014) – Figure 3 **(*)** ; Noma (2017) – Figure 5 ; Gallardo *et al.* (2019) – Figure 1.

Supplementary Figure S1: Spatial organization of fungal chromosomes, as described in the literature. *Examples of 3D models of Neurospora crassa, Saccharomyces cerevisiae and Schizosaccharomyces pombe genomes, found in previously published articles. Notably, these models are either drawings, meaning that they are interpretations of the data made by the authors, or real 3D objects (see black stars), meaning that they arise from the application of dedicated algorithms for the calculation of spatial coordinates, based on Hi-C contact measurements. In both situations, these models summarize current knowledge of the overall organization of genomes of these three species of fungi (see the main text for descriptions of interesting properties).*

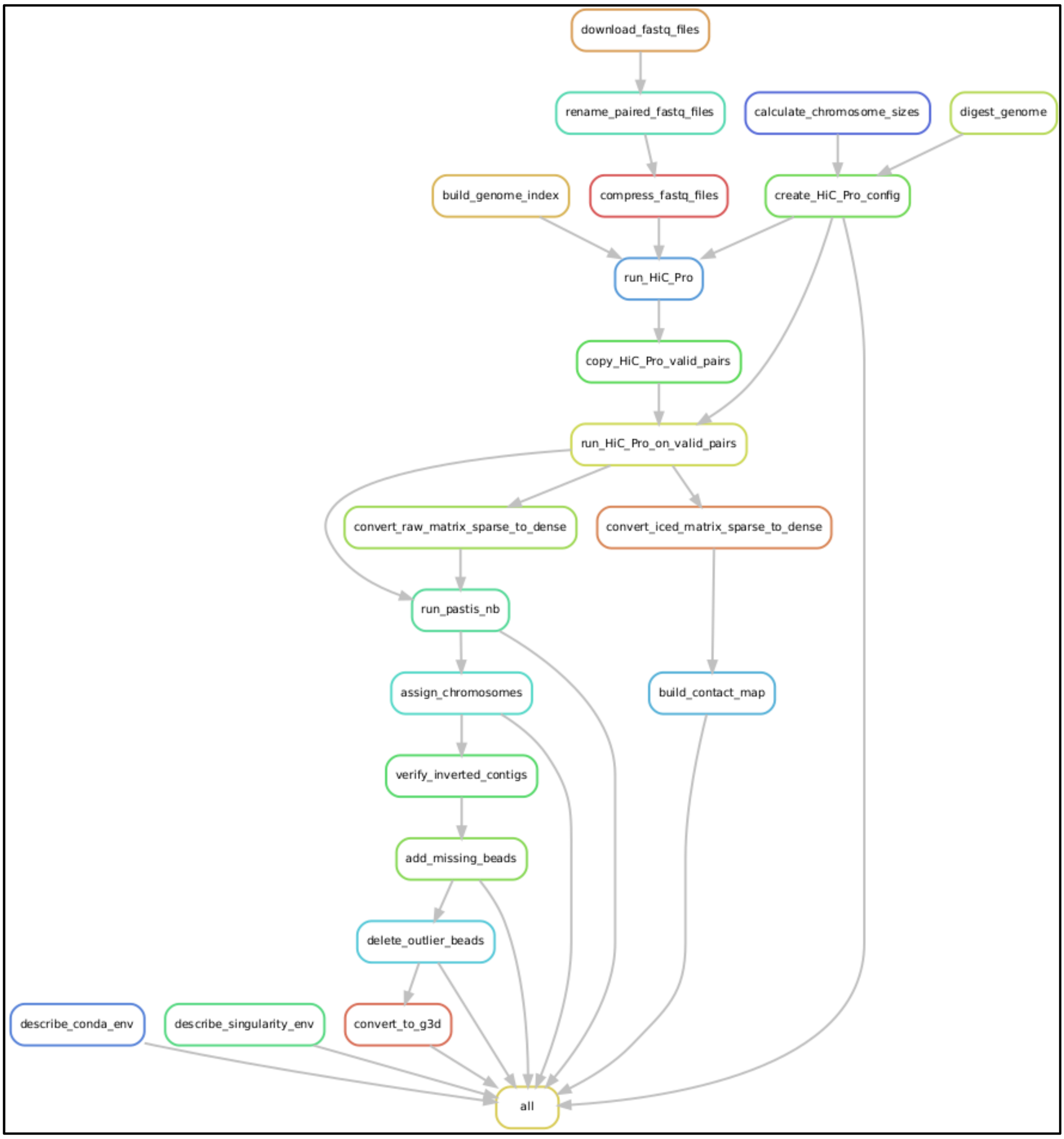

Supplementary Figure S2: Directed acyclic graph of the 3DGB workflow as exported from Snakemake. *Each box refers to a Snakemake rule with defined inputs and outputs. Rules can be run in parallel by Snakemake. More information can be found in https://github.com/data-fun/3d-genome-builder.*

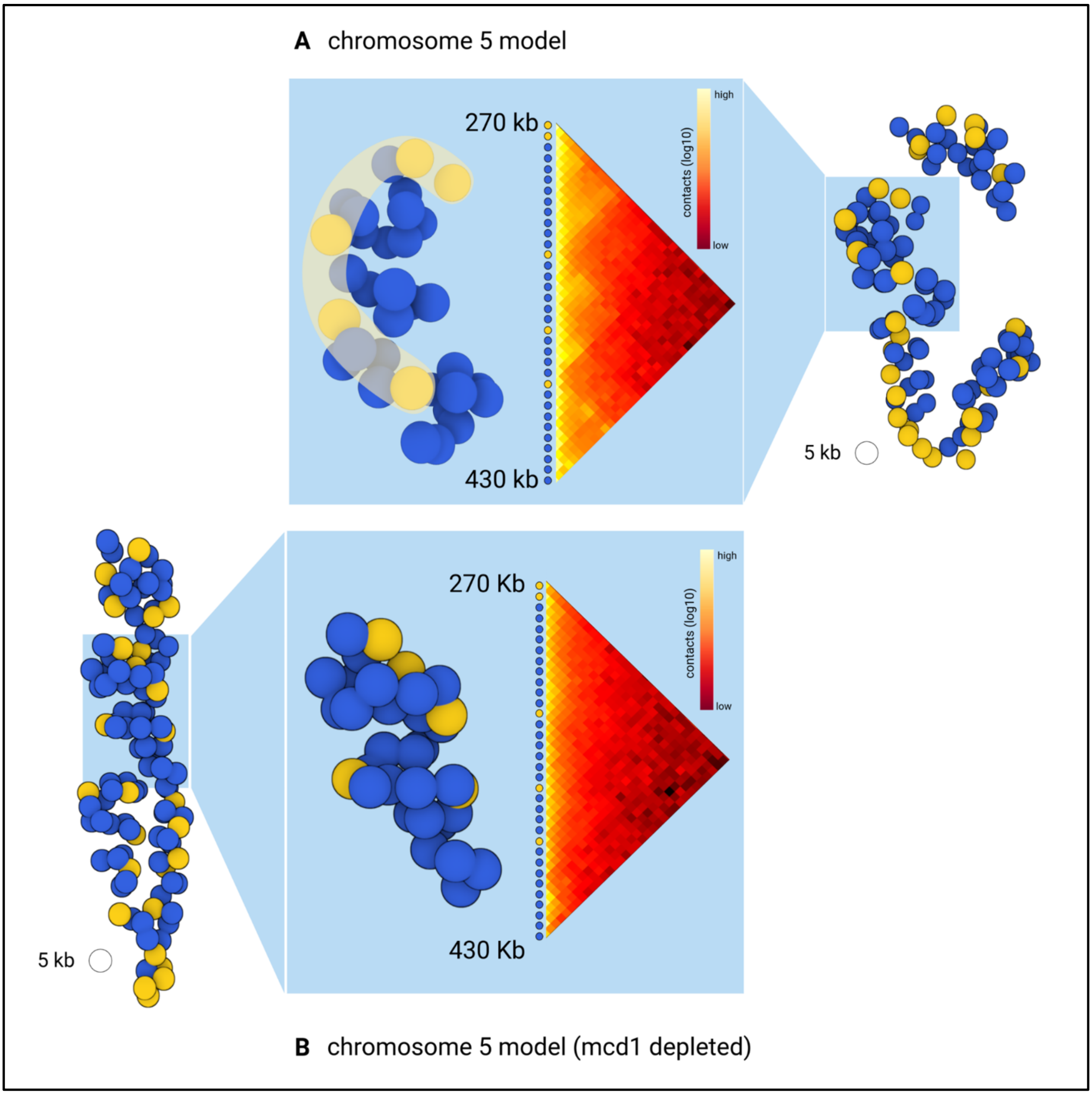

Supplementary Figure S3: 3D skeleton formed by cohesin in* S. cerevisiae *chromatin, example of chromosome 5. (A) *Isolation of chromosome 5 from the overall structure obtained with the wild type strain and* ***(B)*** *isolation of chromosome 5 from the overall structure obtained with the strain depleted for the Mcd1 cohesin subunit. Yellow beads correspond to the backbone of CARs (see the main text), as defined in the wild type situation.*

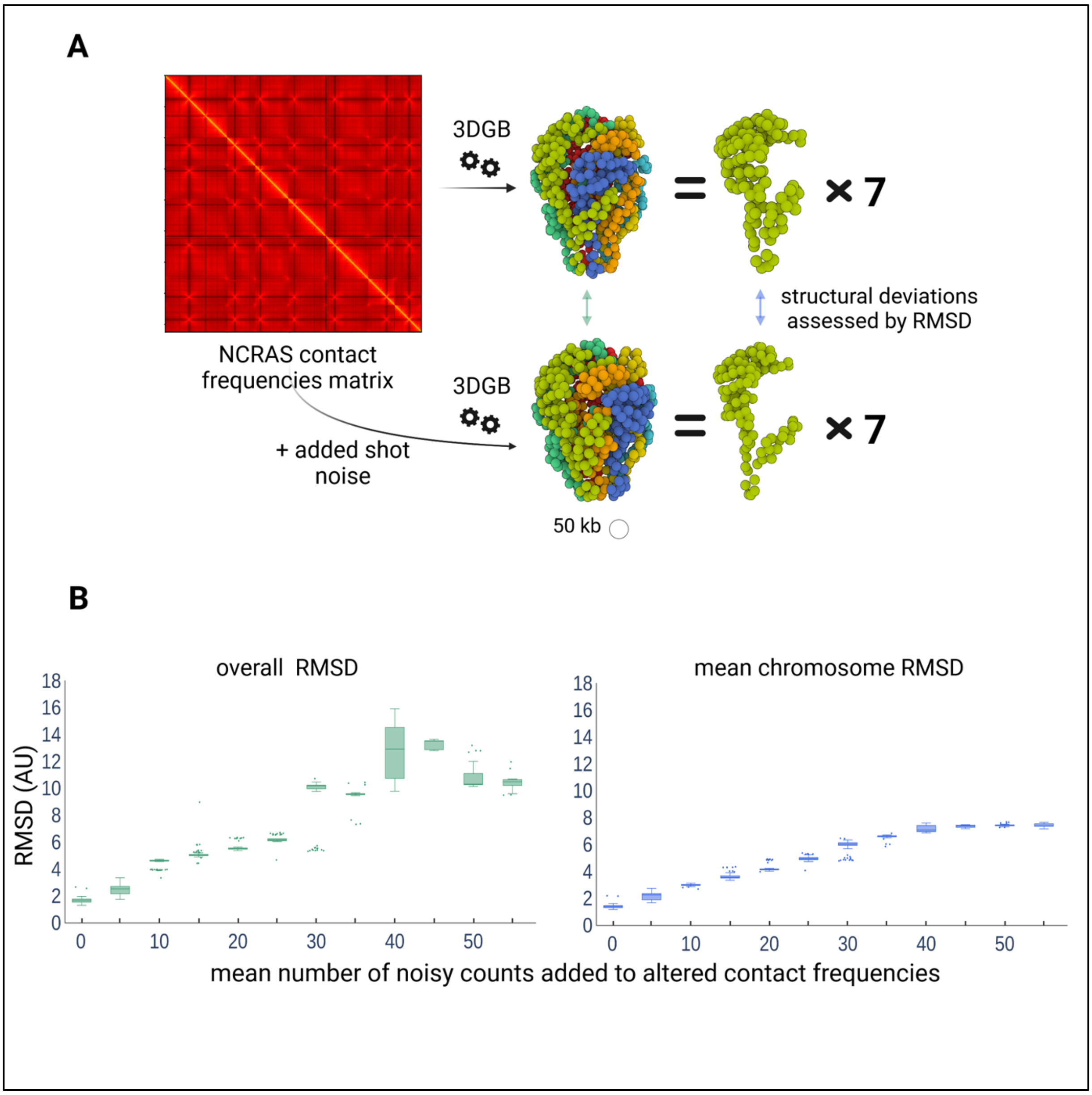

Supplementary Figure S4: Quantification of the stability of 3D models to random noise added to original contact frequencies. (A) *Each pairwise frequency of contacts is randomly modified by adding a count value drawn from a Poisson distribution with a specified mean parameter (see* ***Methods****). 3D models are created with 3DGB (at 50 kb resolution) and compared by calculating RMSD scores.* ***(B)*** *Values of RMSD scores (arbitrary unit) obtained for different values of noise,* i.e. *different values for the mean parameter used in the Poisson distribution. For a given noise value, 50 models are generated, the RMSD score is either calculated on the overall structure composed of all chromosomes (green boxes, on the left) or averaged on the RMSD scores obtained for the 7 individual chromosomes (blue boxes, on the right).*

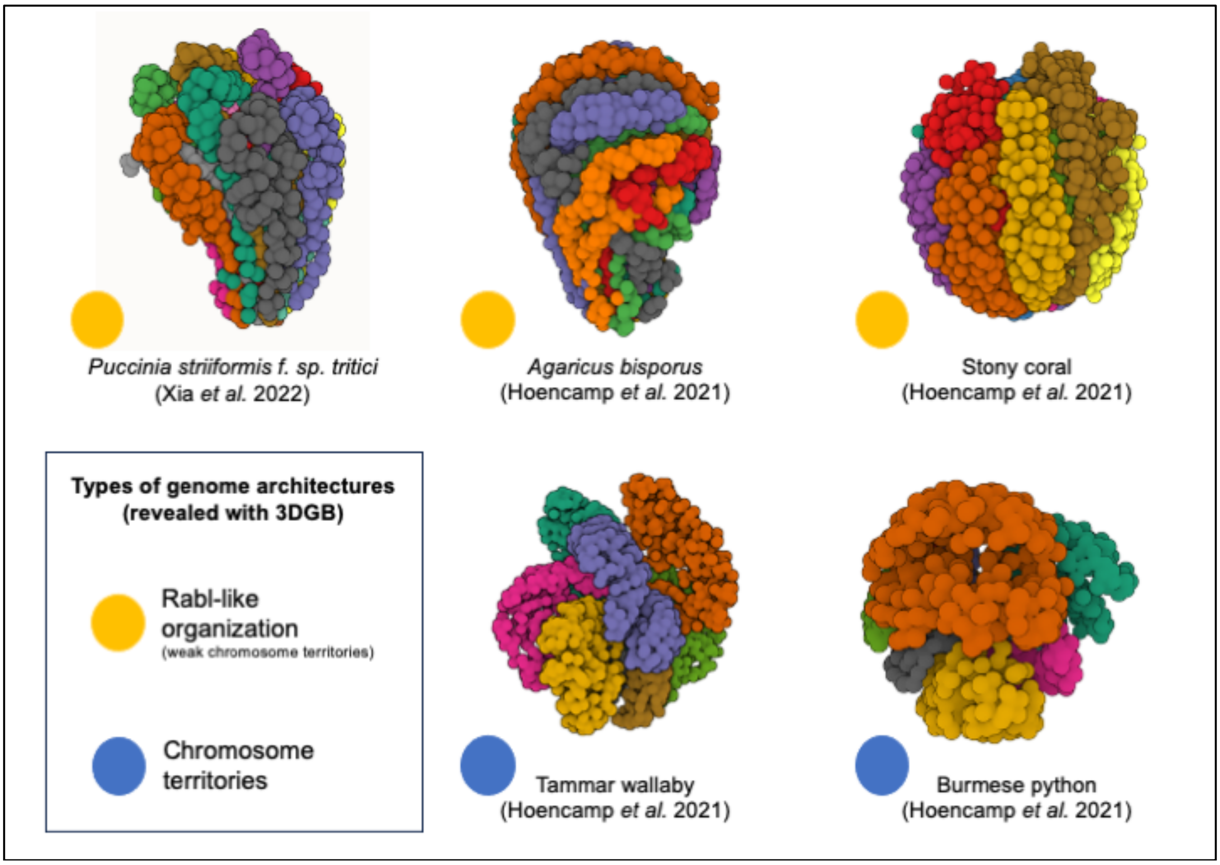

Supplementary Figure S5: Additional models obtained with 3DGB. The species presented here were chosen to reveal two types of genome architectures, as described in Hoencamp et al. (2021) (see the main text). Organization in "Rabl" is shown with a yellow sticker, whereas the organization into canonical chromosome territories is shown with a blue sticker. 3D models were built for two additional fungal species: *Puccinia striiformis f. sp. Tritici*, *i.e.* a fungus causing stripe rust disease on wheat, and *Agaricus bisporus*, *i.e.* a common mushroom widely consumed in the world, and for three non-fungal species: stony coral, tammar wallaby and burmese python. The results obtained are consistent with the literature, revealing the two types of expected genome architectures: a Rabl-like organization (yellow) and a canonical organization into chromosome territories (blue).

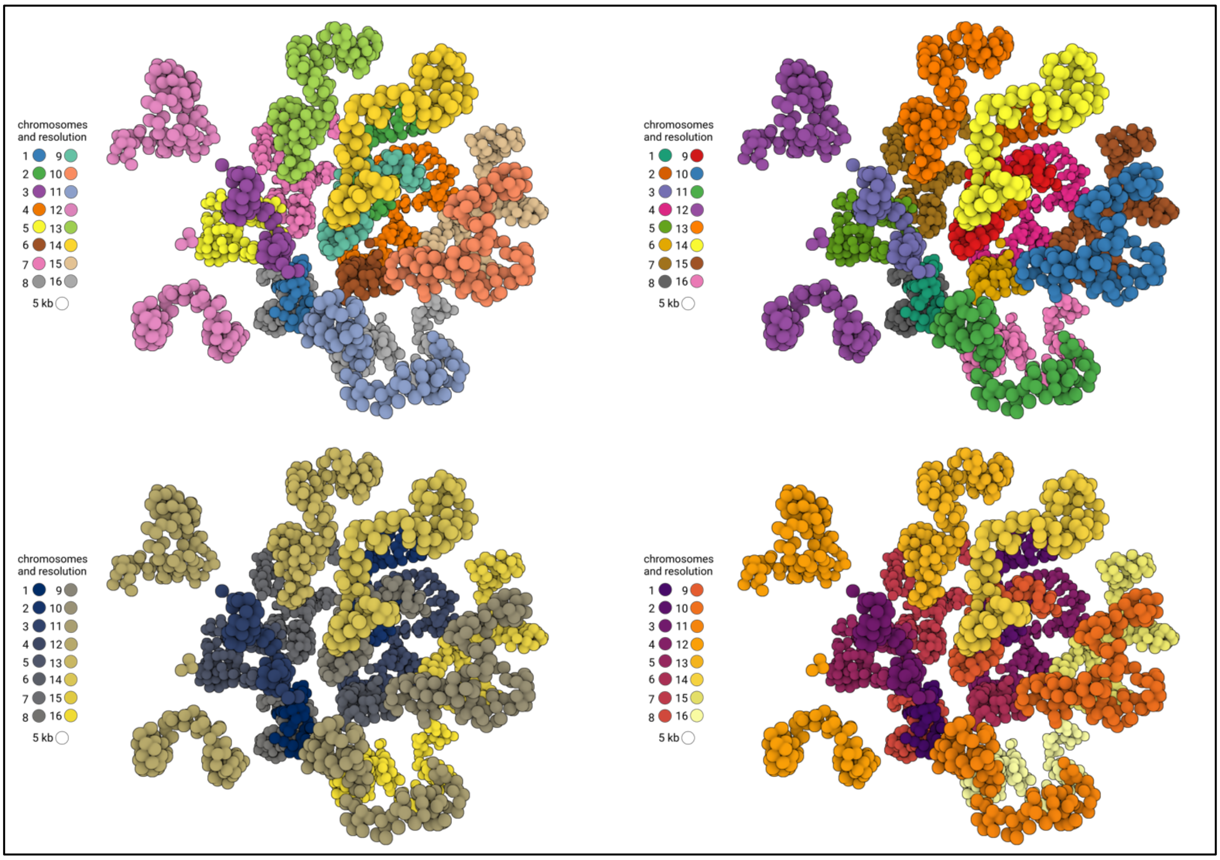

Supplementary Figure S6: Different visualizations of the 3D model of S. cerevisiae chromatin obtained with 3DGB. *Different color scales were used to represent the same 3D model, multiple times. For this the Mol* software is very practical. From the PDB file, the colors can be changed and easily adapted to the user's visual constraints.*
